# Enzyme systems involved in glucosinolate metabolism in *Companilactobacillus farciminis* KB1089

**DOI:** 10.1038/s41598-021-03064-7

**Published:** 2021-12-09

**Authors:** Hiroko Watanabe, Riku Usami, Shigenobu Kishino, Kengo Osada, Yudai Aoki, Hironobu Morisaka, Masatomo Takahashi, Yoshihiro Izumi, Takeshi Bamba, Wataru Aoki, Hiroyuki Suganuma, Jun Ogawa

**Affiliations:** 1 Division of Applied Life Sciences, Graduate School of Agriculture, Kyoto University, Kitashirakawaoiwake-cho, Sakyo-ku, Kyoto, 606-8502 Japan; 2 Laboratory for Circular Bioeconomy Development, Office of Society Academia Collaboration for Innovation, Kyoto University, Kitashirakawaoiwake-cho, Sakyo-ku, Kyoto, 606-8502 Japan; 3 Nature and Wellness Research Department, Innovation Division, KAGOME CO. LTD., 17 Nishitomiyama, Nasushiobara-shi, Tochigi, 329-2762 Japan; 4 Division of Metabolomics, Medical Institute of Bioregulation, Kyushu University, 3-1-1 Maidashi, Higashi-ku, Fukuoka, 812-8582 Japan

**Keywords:** Applied microbiology, Bacteria

## Abstract

Cruciferous vegetables are rich sources of glucosinolates (GSLs). GSLs are degraded into isothiocyanates, which are potent anticarcinogens, by human gut bacteria. However, the mechanisms and enzymes involved in gut bacteria-mediated GSL metabolism are currently unclear. This study aimed to elucidate the enzymes involved in GSL metabolism in lactic acid bacteria, a type of gut bacteria. *Companilactobacillus farciminis* KB1089 was selected as a lactic acid bacteria strain model that metabolizes sinigrin, which is a GSL, into allylisothiocyanate. The sinigrin-metabolizing activity of this strain is induced under glucose-absent and sinigrin-present conditions. A quantitative comparative proteomic analysis was conducted and a total of 20 proteins that were specifically expressed in the induced cells were identified. Three candidate proteins, β-glucoside-specific IIB, IIC, IIA phosphotransferase system (PTS) components (*Cf*PttS), 6-phospho-β-glucosidase (*Cf*PbgS) and a hypothetical protein (*Cf*NukS), were suspected to be involved in sinigrin-metabolism and were thus investigated further. We hypothesize a pathway for sinigrin degradation, wherein sinigrin is taken up and phosphorylated by *Cf*PttS, and subsequently, the phosphorylated entity is degraded by *Cf*PbgS. As expression of both *pttS* and *pbgS* genes clearly gave *Escherichia coli* host strain sinigrin converting activity, these genes were suggested to be responsible for sinigrin degradation. Furthermore, heterologous expression analysis using *Lactococcus lactis* suggested that *Cf*PttS was important for sinigrin degradation and *Cf*PbgS degraded phosphorylated sinigrin.

## Introduction

Isothiocyanates (ITCs) are bioactive functional food factors that exert anti-oxidative, anti-inflammatory and anticarcinogenic effects and induce a host-detoxifying defense system^1–5^. ITCs are derived from glucosinolates (GSLs), which have a non-bioactive thioglucoside bond and are present in these cruciferous vegetables such as broccoli^6^. Their production involves hydrolysis of the *S*-glycosidic linkage in GSLs by human gut bacteria^7^ as common in the metabolism of other glycosides^8^. Thus, with gut bacteria mainly responsible for the beneficial physiological effects of ITCs upon GSL intake, there is a great interest in the determination of how human gut bacteria can degrade GSLs. Some lactic acid bacteria such as *Ligilactobacillus* *agilis* R16^9^ and *Lactococcus lactis* subsp. *lactis* KF147^10^ have exhibited sinigrin (allyl-β-glucosinolate) degradation. Since lactic acid bacteria exist in intestinal tract and in fermented vegetables, they probably play important roles in GSLs-degradation and ITCs-generation in the process of digestion and fermentation, respectively. However, the corresponding enzymes remained to be elucidated.

**Figure 1:**
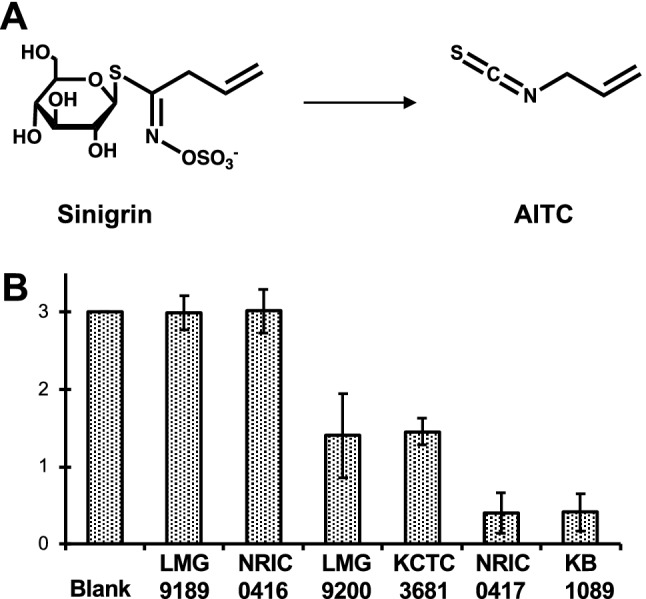
Screening of glucosinolate (GSL)-degrading lactic acid bacteria. (**A**) Structures of the substrate (sinigrin) and product (allyl isothiocyanate, AITC). (**B**) Amounts of residual sinigrin in the culture medium (initial sinigrin; 3.0 mM) after 24 h of cultivation of each strain of *Cb. farciminis*. Blank indicates the uninoculated sample. Data represent mean values (n = 3). Error bars indicate 95% confidence interval (CI).

We isolated *Companilactobacillus farciminis* KB1089 with notable sinigrin-degrading and allylisothiocyanate (AITC)-generating activities (Fig. 1A) from Japanese traditional-vegetable pickles. In this paper, we describe identification of the genes responsible for sinigrin degradation to AITC in *Cb. farciminis* KB1089 by quantitative comparative proteomic analysis. Heterologous expression and functional analysis of these genes first confirmed the involvement of a coupled action by β-glucoside-specific IIB, IIC, IIA phosphotransferase system (PTS) components (*Cf*PttS) and 6-phospho-β-glucosidase (*Cf*PbgS) in sinigrin degradation by lactic acid bacteria.

## Results

### Screening of sinigrin-degrading lactic acid bacteria

We performed screening of sinigrin-degrading bacteria among 279 strains, including plant associated lactic acid bacteria and animal gut bacteria. Only four strains exhibited sinigrin-degrading activity during cultivation in the sinigrin-containing culture medium (S3-mMRS). The lactic acid bacteria strain, KB1089, isolated from pickled Japanese turnips, exhibited the highest sinigrin-degrading activity. This strain was identified as *Companilactobacillus farciminis *via 16S rRNA and *pheS* gene sequencing. Then, sinigrin-degrading activities of another five strains of *Cb. farciminis* available in public culture collections were evaluated. *Cb. farciminis* LMG9200, KCIC3681 and NRIC0417 exhibited sinigrin-degrading activity, whereas LMG9189 and NRIC0416 exhibited no activity (Fig. 1B). Thus, the sinigrin-degrading phenotype appeared to be strain-specific. Strain KB1089 exhibited the highest activity and was used for further investigation.

### Sinigrin-degrading activity of Cb. farciminis KB1089

**Figure 2:**
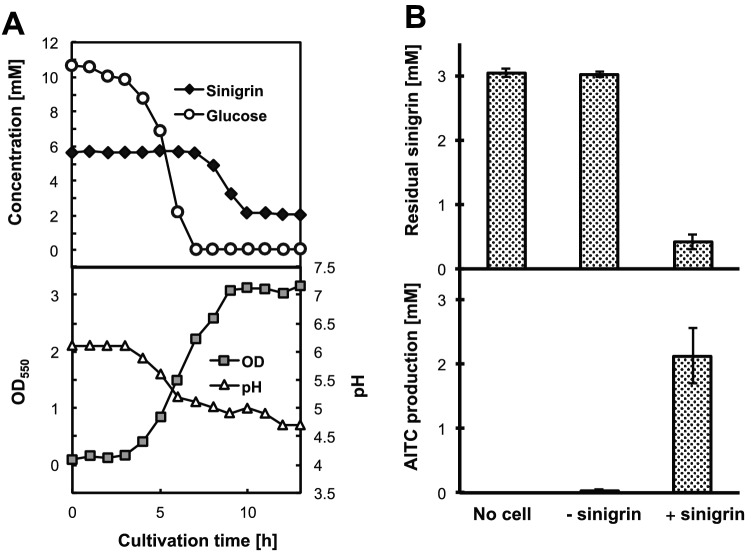
Sinigrin-degrading activity of *Cb. farciminis* KB1089. (**A**) Profiles of glucose and sinigrin concentrations, optical density (OD_550_) and pH during *Cb. farciminis* KB1089 cultivation in the G10S6-mMRS medium. (**B**) Amounts of residual sinigrin (upper graph) and produced AITC (lower graph) in the resting cell reaction mixtures using washed cells obtained after glucose consumption cultivated in G3-mMRS medium (−sinigrin) or G3S3-mMRS medium (+sinigrin). ‘No cell’ indicates a reaction solution without cells. Data represent mean value (n = 3). Error bars indicate 95% CI.

The strain KB1089 degraded sinigrin only after complete glucose consumption coupled with OD increase and pH decline during the cultivation in G10S6-mMRS medium containing sinigrin and glucose (Fig. 2A). The cultivated cells in the sinigrin-containing medium (G3S3-mMRS) showed sinigrin-degrading and AITC-producing activities (Fig. 2B, Supplementary Fig. S1). On the other hand, the cultivated cells in the sinigrin-free medium (G3-mMRS medium) showed no sinigrin-degrading activity (Fig. 2B). This indicated that the sinigrin-degrading activity of *Cb. farciminis* KB1089 was induced by sinigrin.

### Comparative proteomics in sinigrin-induced and non-induced cells of Cb. farciminis KB1089

**Figure 3:**
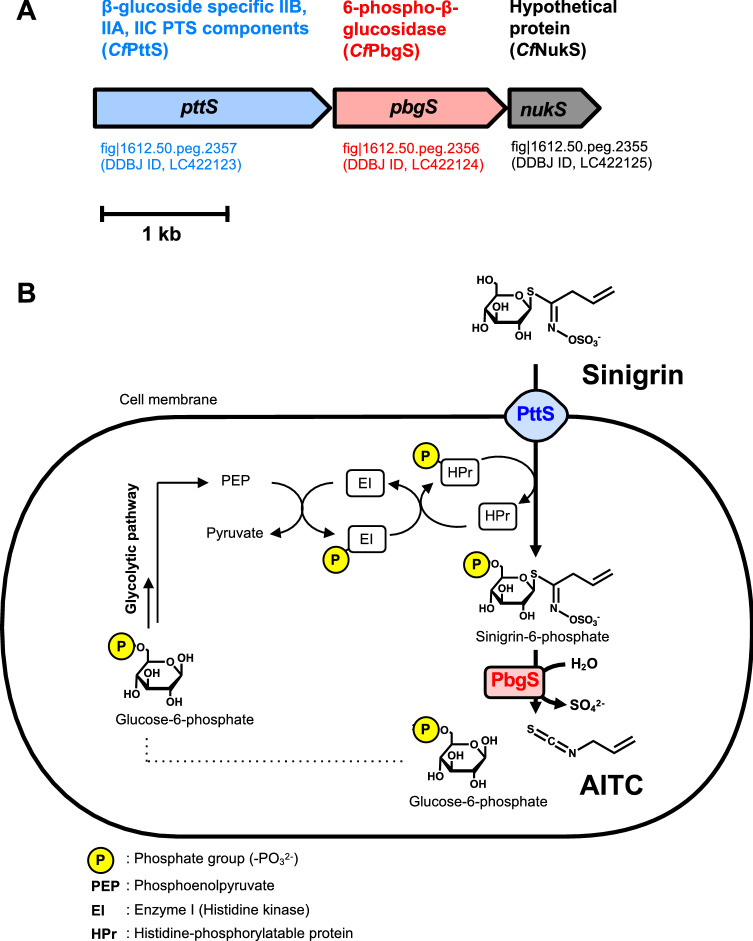
The gene cluster encoding the candidate proteins found in the genome of *Cb. farciminis* KB1089 (**A**) and the putative pathway of sinigrin degradation (**B**).

The proteins expressed in the sinigrin induced cells and in the non-induced cells were digested and applied to nano-liquid chromatography–tandem mass spectrometry (LC–MS/MS). The bottom-up proteomics analysis of the results revealed the amino acid sequences of the sinigrin-induced proteins. A total of 20 proteins were specifically expressed in the induced cells (Table 1). Among the 20 identified proteins, we selected 3 proteins:β-glucoside-specific IIB, IIC, IIA PTS component (*Cf*PttS), 6-phospho-β-glucosidase (*Cf*PbgS) and a hypothetical protein (*Cf*NukS) for further analysis based on the following observations: The three genes fig|1612.50.peg.2357, fig|1612.50.peg.2356 and fig|1612.50.peg.2355 encoding *Cf*PttS, *Cf*PbgS and *Cf*NukS, respectively, existed adjacent to each other in the *Cb. farciminis* KB1089 genome (Table 1, Fig. 3A); The homologous genes were present in the genomes of the other sinigrin-degrading strains of *Cb. farciminis* (strains LMG9200, KCTC3681 and NRIC0417); No homologous genes were found in the genomes of the strains of *Cb. farciminis* without the sinigrin-degrading activity (the strains LMG9189 and NRIC0416).

**Table 1:** Proteins detected only in induced cells of *Companilactobacillus farciminis* KB1089.

Protein accession in the proteomic data set	Coverage	Score Mascot	#PSMs	Gene accession (PATRIC ID)	Gene description
fig|6666666.361936.peg.193	30.0	50.6	3	fig|1612.50.peg.18	Copper-transporting ATPase
fig|6666666.361936.peg.624	9.62	36.6	1	fig|1612.50.peg.198	hypothetical protein
fig|6666666.361936.peg.592	8.51	37.7	1	fig|1612.50.peg.230	Putative regulator of the mannose operon, ManO
fig|6666666.361936.peg.125	24.1	28.5	1	fig|1612.50.peg.450	hypothetical protein
fig|6666666.361936.peg.2171	17.3	42.3	1	fig|1612.50.peg.1020	hypothetical protein
fig|6666666.361936.peg.2179	6.78	32.7	2	fig|1612.50.peg.1028	Hydroxymethylglutaryl-CoA reductase
fig|6666666.361936.peg.1153	4.03	25.0	1	fig|1612.50.peg.1081	Autolysin, amidase
fig|6666666.361936.peg.2477	6.47	33.5	2	fig|1612.50.peg.1254	hypothetical protein
fig|6666666.361936.peg.373	40.7	429.2	24	fig|1612.50.peg.1329	NAD-dependent protein deacetylase of SIR2 family
fig|6666666.361936.peg.372	28.2	216.2	12	fig|1612.50.peg.1330	NAD-dependent protein deacetylase of SIR2 family
fig|6666666.361936.peg.1002	10.7	96.0	3	fig|1612.50.peg.1594	4-hydroxy-tetrahydrodipicolinate synthase
fig|6666666.361936.peg.2091	27.3	88.7	6	fig|1612.50.peg.1699	Regulatory protein Spx
fig|6666666.361936.peg.2480	30.4	28.7	3	fig|1612.50.peg.1810	Thioredoxin
fig|6666666.361936.peg.1539	4.67	26.6	1	fig|1612.50.peg.1854	Ribonuclease HII
fig|6666666.361936.peg.1547	7.53	118.7	2	fig|1612.50.peg.1862	Thymidylate synthase
fig|6666666.361936.peg.1436	16.7	51.7	1	fig|1612.50.peg.2065	SSU ribosomal protein S21p
fig|6666666.361936.peg.1787	2.62	29.8	2	fig|1612.50.peg.2257	ATP synthase F0 sector subunit c
fig|6666666.361936.peg.1336	25.1	405.8	21	fig|1612.50.peg.2355	hypothetical protein
fig|6666666.361936.peg.1335	6.69	104.0	5	fig|1612.50.peg.2356	6-phospho-β-glucosidase
fig|6666666.361936.peg.1333	6.69	38.9	1	fig|1612.50.peg.2357	PTS system, β-glucoside-specific IIB component/PTS system, β-glucoside-specific IIC component/PTS system, β-glucoside-specific IIA component (β-glucoside-specific IIB, IIA, IIC PTS components)

The data were filtered with cut-offs Score Mascot > 0 and q-value ≤ 0.01, corresponding to a 1% false discovery rate on a spectral level. Protein accession numbers are available in the data set deposited in jPOST with accession number PXD011820. The numbers of peptides spectrum matches (#PSMs) reflect approximate amounts of detected proteins. Gene accession numbers corresponded to the detected proteins are available in database provided by the Pathosystems Resource Integration Center (PATRIC). The genes with PATRIC IDs fig|1612.50.peg.2355, fig|1612.50.peg.2356 and fig|1612.50.peg.2357 are selected as candidates.

Protein analysis using InterPro (https://www.ebi.ac.uk/interpro/) revealed the following results: *Cf*PttS contains three structurally distinct domains, IIA, IIB and IIC^11^, which form a membrane-bound complex; *Cf*PbgS belongs to the GH1 family^12^; and *Cf*NukS contains a phosphate-binding loop (P-loop), which is a common motif in ATP-binding proteins^13^ (Table S4). The PTS is a major machinery for importing sugars into the cell with simultaneous phosphorylation of the sugars, which initiate sugar metabolisms in lactic acid bacteria^11^. *Cf*PttS and *Cf*PbgS appeared to be involved in sugar metabolism initiated by the PTS. We hypothesized the existence of a sinigrin-degrading pathway in *Cb. farciminis* KB1089 based on the PTS-mediated pathway (Fig. 3B). In the hypothetical pathway, sinigrin is imported to the cytosol, and its glucose moiety is phosphorylated by *Cf*PttS. The phosphate group may be transferred from phosphoenolpyruvate (PEP) via the universal PTS components, Enzyme I (EI) and histidine-containing phosphocarrier protein (HPr)^11^, and *Cf*PttS to sinigrin. Subsequently, the *S*-glycosidic linkage of phosphorylated sinigrin is hydrolyzed by *Cf*PbgS, followed by a nonenzymatic rearrangement and sulfate group elimination to yield AITC^14^.

### Heterologous expression and functional analysis of candidate proteins

**Figure 4:**
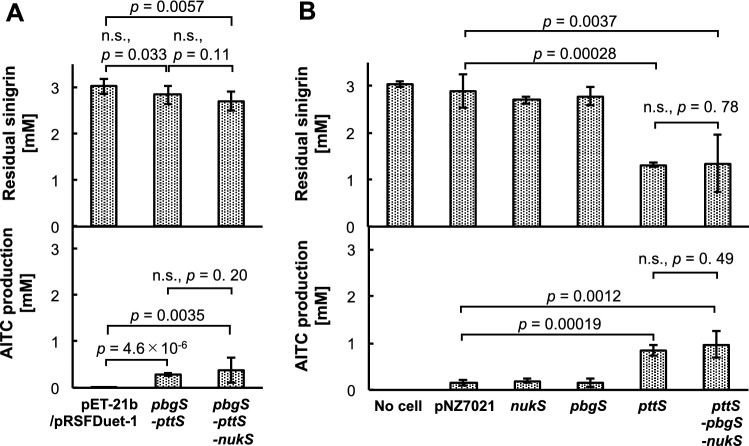
Sinigrin-degrading activity of transformed *E. coli* or *Lc. lactis* in the resting cell reactions using washed cells. (**A**) Amounts of residual sinigrin and produced AITC in the reaction mixtures using transformed *E. coli*. ‘pET-21b/pRSFDuet-1’ indicates the vector control strain; ‘*pbgS-pttS*’ indicates the strain pET21-*pbgS*/pRSFDuet-*pttS* and ‘*pbgS-pttS-nukS*’ indicates the strain pET21-*pbgS*/pRSFDuet-*pttS-nukS.* (**B**) Amounts of residual sinigrin and produced AITC in the reaction mixtures using transformed *Lc. lactis.* ‘No cell’ indicates a reaction solution without cells; ‘pNZ7021’ indicates the vector control strain; ‘*nukS*’ indicates the strain pNZ7021-*nukS*; ‘*pbgS*’ indicates the strain pNZ7021-*pbgS;* ‘*pttS*’ indicates the strain pNZ7021-*pttS*; ‘*pttS*-*pbgS*-*nukS*’ indicates the strain pNZ7021-*pttS*-*pbgS*-*nukS*. Data represent mean values (n = 3)*.* Error bars indicate 95% CI. Student’s t-test was performed for statistical analysis. n.s., no significant difference.

The functions of the genes (*pttS*, *pbgS* and *nukS*) were evaluated through the heterologous expression in *E. coli* Rosetta 2 (DE3) and *Lc. lactis* NZ9000. None of the *E. coli* strains harboring empty vectors showed target activity. The strains harboring one candidate gene (pET28-*pttS*, pET-28-*pbgS*, and pET28-*nukS*), or a combination of two genes, namely, *pttS* and *nukS* (pRSF-*pttS*-*nukS*), or *pbgS* and *nukS* (pET21-*pbgS*/pRSF-*nukS*), showed no target activity. The two *E. coli* transformants that harbored both *pttS* and *pbgS* genes clearly demonstrated sinigrin-degrading activity (i.e. *E. coli* pET21-*pbgS/*pRSFDuet-*pttS*-*nukS* and *E. coli* pET21-*pbgS*/pRSFDuet-*pttS*) (Fig. 4A). These strains exhibited a similar extent of target activity (Fig. 4A). Therefore, we concluded that *Cf*PttS and *Cf*PbgS are probably the enzymes responsible for sinigrin degradation. However, the function of the hypothetical protein (*Cf*NukS) remains to be elucidated.

The *Lc. lactis* NZ9000 transformants harboring only the *nukS* gene (*Lc*. *lactis* pNZ7021-*nukS*) or the *pbgS* gene (*Lc*. *lactis* pNZ7021-*pbgS*) degraded sinigrin into AITC the same extent as *Lc*. *lactis* pNZ7021. However, the strains harboring only the *pttS* gene (*Lc*. *lactis* pNZ7021-*pttS*) and all three candidate genes (*Lc*. *lactis* pNZ7021-*pttS*-*pbgS*-*nukS*) exhibited higher sinigrin-degrading activity (Fig. 4B). These strains exhibited the same extent activities (Fig. 4B).

**Figure 5:**
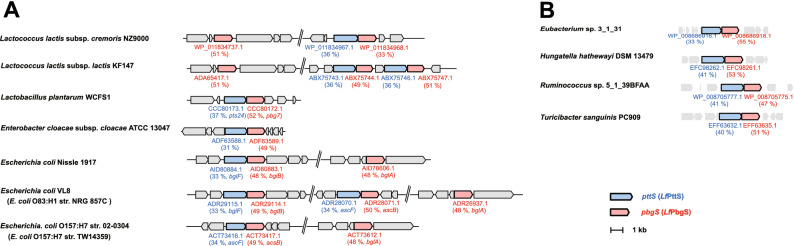
Distribution of gene clusters containing *pttS* and *pbgS* homologues in bacteria. Genes encoding homologues of *Cf*PttS and *Cf*PbgS are indicated in blue and red, respectively. The NCBI accession number assigned to each gene encoding a *Cf*PttS or *Cf*PbgS homologue and its sequence identity obtained by BLASTP analysis as well as its gene symbol are presented below its coding region. Sequence identities are presented in brackets below the coding regions. (**A**) The homologues of *pttS* and *pbgS* in GSL-degrading bacteria. GSL-degrading activities of all bacteria listed above were confirmed in the present study or in previous studies^15–17^. The strains written in parenthesis were used as genome reference in the previous study. (**B**) The homologues of *pttS* and *pbgS* in gut bacteria. All bacteria listed are gut bacteria whose genomic sequences were obtained by metagenomic sequencing of gut microbiome in the Human Microbiome Project^18^.

Two genes encoding *Cf*PbgS homologues were found in *Lc. lactis* NZ9000 through a Basic Local Alignment Search Tool (BLAST) search. One of these genes exists adjacent to a gene encoding a *Cf*PttS homologue (Fig. 5). It is assumed that the sinigrin-degrading activity of the vector control strain is caused by these homologues. Since the exogenous gene expression of *Cf*PttS led to an increase in activity, transportation and phosphorylation have been considered as significant processes for sinigrin degradation in *Lc. lactis* NZ9000.

Furthermore, we obtained phosphorylated sinigrin (Supplementary Fig. S2) using β-glucoside kinase (BglK) from *Klebsiella pneumoniae* ATCC23357, as the phosphorylation activity on the 6ʹ carbon of β-thioglucosides has been previously reported^19^. We evaluated and compared the glucosidase activity of *Cf*PbgS for sinigrin and phosphorylated sinigrin using cell-free extracts of a strain expressing *Cf*PbgS, namely, *Lc. lactis* pNZ7021-*pbgS*. AITC production was detected only in the reactions using phosphorylated sinigrin (Supplementary Fig. S3). These results are consistent with the putative pathway, which assumes that sinigrin is degraded following transport into cells and undergoes simultaneous phosphorylation, which is carried out by *Cf*PttS and *Cf*PbgS, as presented in Fig. 3B.

## Discussion

In this study, we isolated *Cb. farciminis* KB1089 as an important sinigrin-degrading lactic acid bacterium. Moreover, we identified two proteins (*Cf*PttS and *Cf*PbgS) as the enzymes that are responsible for sinigrin degradation. Our results suggest that PTS mediates substrate import and that phosphorylation is involved in the GSL metabolism of *Cb. farciminis* KB1089. To date, three bacterial enzymes or genes associated with GSL metabolism have been identified: from *Citrobacter* WYE1, *E. coli* and *B. thetaiotaomicron* VIP-5482. GH3 family β-*O*-glucosidase was isolated as a GSL-degrading enzyme from the soil isolate *Citrobacter* WYE1^20^. Another enzymatic mechanism that involves four gene products in *B. thetaiotaomicron* VIP-5482 has been proposed^21^. The metabolic pathway is initiated by the NAD^+^-dependent oxidation of the sugar moiety, which is followed by hydrolysis and concomitant reduction using NADH^21^. In addition, it was also found that 6-phospho-β-glucosidase is involved in GSL metabolism in *E. coli* strain O157:H7^22^. By differential proteomics on *E. coli* VL8, a glucose-specific PTS was shown to be induced by sinigrin, which provides some evidence that a phosphorylation step is important for the GSL hydrolysis^23^. These reports indicate that phosphorylation of the glucose moiety through PTS is required for GSL metabolism in these *E. coli* strains. Thus, the GSL-metabolizing mechanism of *Cb. farciminis* KB1089 appears to be similar to that of *E. coli* rather than *Citrobacter* WYE1 and *B. thetaiotaomicron* VIP-5482.

In lactic acid bacteria, some PTS transporters for sugar substrates, such as monosaccharide, disaccharide and sugar alcohol, were found. Moreover, PTS-mediated metabolic pathways of glycosylated aromatic compounds (amygdalin, esculin and salicin) in *Lb. acidophilus* NCFM have been recently reported^24^. *Lb. acidophilus* NCFM metabolizes β-(1,6)-diglucoside and β-glucoside consisting of mono- or bicyclic aromatic rings conjugated with a glycosyl moiety via PTS, but not sinigrin (allyl-β-thioglucoside)^24^. This enzymatic mechanism appears to be similar to that of sinigrin degradation catalyzed by *Cf*PttS and *Cf*PbgS, whereas the target specificity (i.e., gene regulation and substrate recognition) appears to be different.

InterPro analysis revealed that the *Cf*PttS consists of three components (IIA, IIB and IIC) (Table S4). The amino acid sequence exhibited 41% identity with the β-glucoside-specific IIABC component (encoded by LBA0275) in *Lb. acidophilus* NCFM^24^, which contributes to the metabolism of plant glucoside that has small aromatic aglycones. *Cf*PttS does not exhibit high global homology with the *N*-acetyl glucosamine-specific PTS component IIBCA or a glucose-specific PTS component in *E. coli* VL8^23^ that has been suspected to be involved in GSL metabolism, whose coding gene is observed in the genomes of the other GSL-degrading strains, O157:H7 and Nissle1917^10,22^. According to PTS-mediated carbohydrate-metabolizing mechanisms, the *Cf*PttS is assumed to enable the transfer of a phosphoryl group to sinigrin from versatile PTS components (EI and HPr). Appropriate arrangement of these components seems important for a successful phosphoryl relay. Destruction of this arrangement by membrane disruption may cause the loss of the sinigrin-degrading activity of cell-free extracts, as observed in *Cb. farciminis* KB1089 in this study and in other GSL-degrading bacteria in previous studies^9,10^.

*Cf*PbgS belongs to the GH1 family (Table S4), which also contains plant and aphid myrosinase^25–27^ and the 6-phospho-β-d-glucosidases (encoded in *bglA* and *ascB*) of *E. coli* O157:H7^22^. *Cf*PbgS exhibits high identities with BglA and AscB of *E. coli* O157:H7 (48% and 50%, as presented in Fig. 5). However, *Cf*PbgS has a low identity (< 20%) with a bacterial myrosinase from *Citrobacter* WYE1, which belongs to the GH3 family^20^. Putative myrosinase activity by the 6-phospho-β-glucosidase from *E. coli* O157:H7, together with the GSL-induced activation of a glucose PTS in *E. coli* VL8^23^, has led to the suggestion that glucose moiety phosphorylation may be a prerequisite for GSL hydrolysis in these *E. coli* strains and in *Cb. farciminis* KB1089.

We found gene clusters containing homologues of *pttS* and *pbgS* in the genomes of other GSL-degrading bacteria^15–17^, including *E. coli* strains isolated as human gut bacteria and human associated bacteria (Fig. 5A). However, no homologues were observed in *B. thethaiotaomicron*^21^. Furthermore, the homologue of *pttS* and *pbgS* were found in other gut bacteria, whose genomes obtained by metagenomic sequencing of gut microbiome in the Human Microbiome Project^18^, such as *Eubacterium* sp. 3_1_31 and *Hungatella hathewayi* DSM 13479 which had not yet been reported as GSL-degraders (Fig. 5B). For further understanding of GSL metabolism in gut bacteria and lactic acid bacteria, a functional analysis of these homologous genes is underway. To the best of our knowledge, this is the first study that identified novel proteins involved in GSL degradation in lactic acid bacteria.

Together with this finding, proteome analysis also revealed that two NAD-dependent deacetylases, enzymes that catalyze protein deacetylation, are strongly induced in sinigrin containing medium (Table 1). Protein acetylation/deacetylation is a hot topic in recent years because it is an evolutionarily conserved post-translational modification that affects enzyme activity, metabolic flux, and many important biochemical processes, but the regulatory mechanisms have not been well characterized in bacteria^28^. Focusing on these deacetylases may lead to a better understanding of the response mechanisms of lactic acid bacteria to the presence of bioactive phytochemicals such as sinigrin or AITC.

## Methods

### Chemicals

Sinigrin was purchased from Sigma-Aldrich (St. Louis, MO, USA) and AITC from Wako Pure Chemicals (Osaka, Japan). All the other chemicals utilized in this study were of analytical grade and are commercially available.

### Culture media

De Man, Rogosa and Sharpe (MRS) broth (Difco, BD, Franklin Lakes, NJ, USA) containing 2% dextrose and basal solution (1% proteose peptone No. 3; 1% beef extract; 0.5% yeast extract; 0.1% polysorbate 80; 0.2% ammonium citrate; 0.5% sodium acetate; 0.01% magnesium sulphate; 0.005% manganese sulphate; 2% dipotassium phosphate) were adjusted to pH 6.5 with HCl and used for lactic acid bacteria cultivation. Four modified MRS (mMRS) media (G10S6-mMRS, S3-mMRS, G3S3-mMRS, and G3-mMRS) were utilized to analyze the activity of lactic acid bacteria on sinigrin degradation. G10S6-mMRS contained 10 mM glucose, 6 mM sinigrin, and the basal solution. S3-mMRS contained 3 mM sinigrin and the basal solution. G3S3-mMRS contained 3 mM glucose, 3 mM sinigrin, and the basal solution. G3-mMRS contained 10 mM glucose and the basal solution. For the cultivation of *E. coli* transformants, Luria–Bertani (LB) medium containing 1% tryptone, 0.5% yeast extract, and 1% NaCl was used. For the cultivation of *Lc. lactis* transformants, modified M17 (mM17) medium obtained by supplementation of 30 mM glucose and 0.1 mg mL^−1^ catalase (from bovine liver) to M17 broth (Difco, BD, Franklin Lakes, NJ, USA) was utilized.

### Bacterial strains, plasmids, and primers

The bacterial strains, plasmids, and primers utilized in this study are presented in Supplementary Tables S1–S3.

### Screening of GSL-metabolizing lactic acid bacteria

Glycerol stocks of laboratory stock lactic acid bacteria, isolated from human feces, animal feces or pickles were inoculated in 10 mL of MRS broth and were cultivated at 30 °C without shaking for 24 h. Culture medium (100 μL) was added to 10 mL of fresh MRS broth and was cultivated at 30 °C without shaking for 24 h. Moreover, culture medium (50 μL) was added to 5 mL of S3-mMRS medium and further cultivated at 30 °C without shaking for 120 h. The culture medium was centrifuged at 12,000× *g* for 20 min, and 600 μL of the obtained supernatant was transferred to a 1.5-mL plastic tube. An identical volume of 30% (w/v) trichloroacetic acid (TCA) solution was added to the tube, mixed by inversion, placed on ice for 30 min and then centrifuged at 12,000× *g* for 20 min. The obtained supernatant was filtered with a membrane filter with a pore size of 0.22 μm and analyzed by high-performance liquid chromatography (HPLC). For detailed analytical conditions of HPLC analysis see Supplementary Materials and Methods.

### Bacterial species identification

Selected strain KB1089 was identified by 16S rRNA and phenylalanyl-tRNA synthetase (*pheS*) gene^29^ sequencing. The detailed methods were provided in Supplementary Materials and Methods.

### Time-course evaluation of bacterial growth, pH, glucose and sinigrin concentration in Cb. farciminis KB1089 culture

*Cb. farciminis* KB1089 was inoculated from a glycerol stock into 5 mL of MRS broth, cultivated at 30 °C without shaking for 24 h, and subcultured in fresh MRS broth under the same condition for 24 h. The subculture medium (350 μL) was inoculated into 35 mL of G10S6-mMRS medium and cultivated at 30 °C without shaking. Then, 1.2 mL of the culture was sampled at different time points, of which 100 μL aliquots were utilized to measure the optical density at 550 nm (OD_550_) and to determine pH using a portable pH meter (Model B212, Horiba, Kyoto, Japan). The remaining 1 mL of culture was centrifuged and treated with TCA solution, as described above, after which the concentrations of sinigrin and glucose were determined via HPLC and using a Biosensor BF-5i (Oji Scientific Instruments, Amagasaki, Japan), respectively. The experiments were performed in triplicate, and the averages of three separate experiments that were reproducible within 10% were presented in Fig. 2A.

### Cloning of nukS, pbgS, and pttS in E. coli and Lc. lactis

Detailed methods for preparation of genomic DNA, extraction of plasmid DNA, DNA sequence analysis, PCR amplification, and purification of PCR products and cloning of *nuS*, *pbgS* and *pttS* in *E. coli* and in *Lc. lactis* were provided in Supplementary Materials and Methods.

### Preparation of washed cell pellets

For preparation of washed cells of *Cb. farciminis* KB1089, a glycerol stock was inoculated into 10 mL of MRS broth in glass test tubes (16.5 × 125 mm), and was cultivated at 28 °C with shaking (120 strokes min^−1^) for 16 h. Furthermore, 100 μL of the culture was added to 10 mL of either G3S3-mMRS or G3-mMRS medium and cultivated at 28 °C with shaking (120 strokes min^−1^) for 7 h. After confirming glucose consumption in the culture medium using a Glucose CII-Test Wako Kit (Wako Pure Chemicals Ind., Osaka, Japan), cells were harvested by centrifugation at 1500 × *g* for 10 min, and pellets were washed twice with 0.85% (w/v) NaCl.

For preparation of washed cells of *E. coli* transformants, they were grown at 37 °C with shaking at 150 strokes min^−1^ in 5 mL of LB medium containing 34 μg mL^−1^ of chloramphenicol and antibiotics corresponding to the resistance-conferring genes present in the indicated harbored vectors (30 μg mL^−1^ of kanamycin for *E. coli* pET28-*nukS*, *E. coli* pET28-*pbgS*, *E. coli* pET28-*pttS* and *E. coli* pRSF-*pttS*-*nukS*, and 100 μg mL^−1^ ampicillin together with 30 μg mL^−1^ kanamycin for *E. coli* pET21-*pbgS*/pRSF-*pttS* and *E. coli* pET21*-pbgS*/pRSF-*pttS*-*nukS*, respectively) in 12 mL culture tubes (Thermo Fisher Scientific, Waltham, MA, USA). About 50 μL of each overnight culture was added to 5 mL of LB medium containing the appropriate antibiotics. Isopropyl β-d-1-thiogalactopyranoside (IPTG) was added to a final concentration of 0.1 mM upon incubation at 37 °C with shaking at 150 strokes min^−1^ for 2.5 h, and cells were cultivated at 20 °C with shaking at 120 strokes min^−1^ for 16 h. The cells were harvested by centrifugation at 1500× *g* for 10 min, washed twice with 0.85% (w/v) NaCl.

For preparation of washed cells of *Lc. lactis* transformants, they were grown under anaerobic conditions (using the AnaroPack, Mitsubishi Gas Chemical, Tokyo, Japan) at 30 °C for 18 h in 5 mL of mM17 medium supplemented with 10 μg mL^−1^ of chloramphenicol in glass test tubes (16.5 × 150 mm). About 250 μL of each culture medium was added to 10 mL of an identical fresh medium. After incubation (under anaerobic conditions at 30 °C for 7 h), the cells were harvested by centrifugation at 1500× *g* for 20 min, washed twice with 0.85% (w/v) NaCl.

### Resting cell reactions using washed cells of Cb. farciminis KB1089, E. coli, and Lc. lactis transformants

The washed cell pellets were resuspended in 1 mL of reaction solution containing 3 mM sinigrin in 20 mM potassium phosphate buffer (KPB, pH 6.5) and incubated at 28 °C with shaking (120 strokes min^−1^) for 16 h. The reaction mixtures were centrifuged at 1500× *g* for 10 min. To detect sinigrin, the supernatants were analyzed by HPLC. Conversely, to detect AITC, 0.6 mL of the supernatants was transferred to a glass test tube, and an identical volume of hexane, including 25 nM benzyl propionate as the internal standard, was added to the sample. The sample was then mixed by vortexing for 1 min and centrifuged at 1500× *g* for 10 min. The aliquot corresponding to the hexane layer was filtered with a membrane filter (Merck Millipore, Darmstadt, Germany) with 0.45 μm pore size and was analyzed by gas chromatography–mass spectrometry (GC–MS). For detailed analytical conditions of GC–MS analysis see Supplementary Materials and Methods.

### Synthesis and analysis of sinigrin-6-sinigrin

Detailed methods of enzymatic synthesis of sinigrin-6-phosphate by BglK, extraction of phosphorylated sugar compounds and detection of phosphorylated sinigrin by high-performance ion chromatography–high-resolution tandem mass spectrometry (HPIC–HRMS/MS) analysis were provided in Supplementary Materials and Methods.

### Reaction using the cell-free extracts of Lc. lactis transformants

Washed cells of *Lc. lactis* pNZ7021-*pbgS* were suspended in 1 mL of 100 mM MES (pH 6.5) and disrupted using Insonator 201 M Ultrasonic Oscillator (Kubota, Osaka, Japan) at 9 kHz, 180 W, 0 °C for four cycles of 5 min each. The supernatants obtained following the removal of cell debris by centrifugation at 8000× *g* for 20 min were used as the cell-free extracts. The cell-free extracts (450 μL) were mixed with 50 μL of 0.3 mg mL^−1^ Ba^2+^ salts containing phosphorylated sugar compounds, obtained as described in Supplementary Materials and Methods, in 100 mM MES (pH 6.5) and incubated at 28 °C with shaking at 120 strokes min^−1^ for 1.5 h. Subsequently, the produced AITC was extracted and analyzed as described above.

### Draft genome sequencing

Draft genome sequences of *Cb. farciminis* KB1089, LMG9189, NRIC0416 and NRIC0417 were determined by the whole-genome shotgun strategy using 454 pyrosequencing in the GS Junior Benchtop System (Roche, Basel, Switzerland). The draft genome sequences of *Cb. farciminis* KB1089, LMG9189, NRIC0416 and NRIC0417 were obtained with 67, 67, 112 and 166 contigs, respectively.

### Quantitative proteomic analysis

Washed cells of *Cb. farciminis* KB109 prepared using G3S3-mMRS and G3-mMRS media, as described above, were used for proteomics as induced and non-induced cells, respectively. For detailed preparation procedures for quantitative proteomic analysis see Supplementary Materials and Methods. LC–MS/MS analysis was conducted using an LC (Ultimate 3000; Thermo Scientific, Waltham, MA, USA) –MS–MS (LTQ Velos Mass Spectrometer, Thermo Scientific, Waltham, MA, USA) system equipped with a long monolithic capillary column. Tryptic digests were separated by reversed-phase chromatography using a monolithic silica capillary column (200 cm long, 0.1 mm ID)^30^ at the flow rate of the two eluents: eluent A, 0.1% (v/v) formic acid; eluent B, 80% acetonitrile containing 0.1% (v/v) formic acid. The gradient started with 5% eluent B, increased to 45% eluent B for 600 min, further increased to 95% eluent B to wash the column for 140 min, returned to the initial condition, and then held for re-equilibration of the column. Separated analytes were detected using a mass spectrometer with a full-scan range of 350–1500 m/z. The analysis program was set to automatically analyze the top 10 most intense ions observed in the MS scan for data-dependent acquisition. An electrospray ionization voltage of 2.4 kV was directly applied to the LC buffer end of the chromatography column by using a MicroTee (Upchurch Scientific, Lake Forest, IL, USA). The ion transfer tube temperature was set to 300 °C. Then, data analysis was conducted using Proteome Discoverer version 2.1 (Thermo Scientific, Waltham, MA, USA). The data were filtered with a cut-off q-value ≤ 0.01, corresponding to a 1% false discovery rate on a spectral level. Protein identification was performed using the Mascot algorithm against the protein database of *Cb. farciminis* KB1089 obtained from draft genome sequence.

## Supplementary Information

Supplementary Information.

## Data Availability

The genome nucleotide sequence data of the *Cb. farciminis* strains are available in the DNA Data Bank of Japan (DDBJ) database under the accession numbers BHYW01000001−BHYW01000067 for KB1089, BHYX01000001−BHYX01000067 for LMG9189, BHYY01000001−BHYY01000112 for NRIC0416, and BHYZ01000001−BHYZ01000166 for NRIC0417. The genomic features database of *Cb. farciminis* KB1089 are available in the PATRIC (the Pathosystems Resource Integration Center) database with Genome ID 1612.50. The proteomic data were deposited in the Japan ProteOme Standard Repository/Database (jPOST)^31^ with the accession number PXD011820. The *pttS*, *pbgS* and *nukS* gene sequences were deposited in DDBJ under accession numbers LC422123, LC422124 and LC422125, respectively.
