# Supplementary material for: Enzyme systems involved in glucosinolate metabolism in Companilactobacillus farciminis KB1089
Source: Sci Rep. 2021 Dec 9;11:23715. doi: 10.1038/s41598-021-03064-7 (PMC8660893; doi:10.1038/s41598-021-03064-7)
Supplement: Supplementary file 1 — Supplementary Information. [file 41598_2021_3064_MOESM1_ESM.docx]

**Supplementary Information**

**Enzyme systems involved in glucosinolate metabolism in *Companilactobacillus farciminis* KB1089**

**Hiroko Watanabe^1,2†^, Riku Usami^1†^, Shigenobu Kishino^1†^, Kengo Osada^1^, Yudai Aoki^3^, Hironobu Morisaka^1^, Masatomo Takahashi^4^, Yoshihiro Izumi^4^, Takeshi Bamba^4^, Wataru Aoki^1^, Hiroyuki Suganuma^3^ and Jun Ogawa^1*^**

^1^Division of Applied Life Sciences, Graduate School of Agriculture, Kyoto University, Kitashirakawaoiwake-cho, Sakyo-ku, Kyoto 606-8502, Japan.

^2^Laboratory for circular Bioeconomy Development, Office of Society Academia Collaboration for Innovation, Kyoto University, Kitashirakawaoiwake-cho, Sakyo-ku, Kyoto 606-8502, Japan.

^3^Health Claim Group, Nature & Wellness Research Department, Innovation Division, Kagome Co. Ltd., 17 Nishitomiyama, Nasushiobara-shi, Tochigi 329-2762, Japan.

^4^Division of Metabolomics, Medical Institute of Bioregulation, Kyushu University, 3-1-1 Maidashi, Higashi-ku, Fukuoka 812-8582, Japan.

*Corresponding author. **^†^**These authors equally contributed to this work.

**Correspondence:** Jun Ogawa, Division of Applied Life Sciences, Graduate School of Agriculture, Kyoto University, Kitashirakawaoiwake-cho, Sakyo-ku, Kyoto 606-8502, Japan.

Tel: +81-75-735-6115, FAX: +81-75-6113, Email: ogawa.jun.8a@kyoto-u.ac.jp

**This file includes:**

Supplementary Materials and Methods

Supplementary References

Figs. S1 to S3

Tables S1 to S4

**Supplementary Materials and Methods**

**HPLC analysis**

Using a Shimadzu LC 10A system (Shimadzu, Kyoto, Japan) equipped with a Cosmosil column (5C_18_-MS-II; 3.0 mm ID × 250 mm; Nacalai Tesque, Kyoto, Japan), the treated mixtures (10 μL) were analyzed by HPLC. The mobile phase for HPLC analysis of the culture supernatants was methanol-H_2_O (20:80, by volume, supplemented with 0.05% [w/v] trifluoroacetic acid). The mobile phase for HPLC analysis of the supernatants of the resting cell reaction mixtures was acetonitrile-H_2_O (7:93, by volume, supplemented with 0.05% [w/v] trifluoroacetic acid). In addition, the column temperature was maintained at 35°C. The flow rate was 1.0 mL min^−1^, and the effluent was monitored using SPD-10A (Shimadzu, Kyoto, Japan) at a wavelength of 235 nm.

**16S rRNA and phenylalanyl-tRNA synthetase (*pheS*) gene sequencing**

The primers used for the 16S rRNA and *pheS* gene sequencing are presented in Table S3. The 16S rRNA gene was amplified by PCR using the primers 27F/1525R and AmpliTaq Gold PR Master Mix (Applied Bioscience, Bedford, MA, USA). The PCR product was gel-purified and sequenced using 27F, 1525R, 516F, 1087F, 531R, 1104R, and 518R (Tsukuba Oligo Service, Tsukuba, Japan). Then, the obtained sequences were assembled using BioEdit to create a contig, which was then searched using BLAST search on the DNA Data Bank of Japan (DDBJ) database.

The *pheS* gene^29^ was amplified by PCR using the primers pheS-21F/pheS-22R (Eurofins Genomics, Luxembourg, Luxembourg). The PCR product was gel-purified and sequenced using the same primers. The obtained sequences were assembled as described above.

**GC–MS analysis**

A treated sample (hexane solution) was analyzed by GC–MS using a GC-MS QP2010 (Shimadzu, Kyoto, Japan) with a GC-2010 gas chromatograph equipped with a split injection system and a Supelco column (SPB-1; 0.25 mm ID × 30 m; Sigma-Aldrich, St. Louis, MO, USA). The column temperature was maintained at 80 °C for 35 min. Moreover, the injector was operated at 250°C. Helium (0.62 mL min^-1^) was used as a carrier gas. MS was performed in the electron impact mode at 70 eV with at a source temperature of 250°C.

**Sample preparation for quantitative proteomic analysis**

Cells in 500 mL cultures of *Cb. farciminis* KB1089, grown either in G3S3-mMRS or G3-mMRS medium, were harvested by centrifugation at 1,500 × *g* for 10 min. The compositions of G3S3-mMRS and G3-mMRS media were shown in the Methods section (Culture media). Cell pellets (0.1 g) were resuspended in 500 μL of lysis buffer (containing 50 mM tris(hydroxymethyl)aminomethane-HCl [pH 7.0], 7 M urea, 2 M thiourea, 2% [w/v] 3-(3-cholamidopropyl)dimethylammonio-1-propanesulfonate, 10 mM dithiothreitol, and 1% [v/v] protease inhibitor cocktail for cell lysis [Sigma-Aldrich, St. Louis, MO, USA]). Cells were disrupted with sonication using a Powersonic model 50 probe sonicator (Yamato Scientific, Tokyo, Japan) and then centrifuged at 14,000 × *g* for 10 min. The resultant supernatant was filtered using an Amicon Ultra-0.5 Centrifugal Filter Unit (10 kDa, EMD Millipore, Billerica, MA, USA) and buffer-exchanged with 0.2 M triethylammonium bicarbonate (TEAB). About 50 μL of tris(2-calboxyethyl) phosphine (0.2 M) was added to the solution; then, the solution was incubated at 55°C for 60 min to reduce proteins. Another 5 μL of 375 mM iodoacetoamide was added, and the solution was incubated at room temperature for 30 min in the dark. Proteins were precipitated by adding 2 mL of ice-cold acetone and then incubated overnight incubation at −20°C. The precipitated proteins were resuspended in 200 μL of 0.2 M TEAB supplemented with 20 μg of sequencing grade modified trypsin (Promega, Madison, WS, USA) and incubated overnight at 37°C. The digested samples were subjected to proteomic analysis.

**Preparation of genomic DNA, extraction of plasmid DNA, DNA sequence analysis, PCR amplification, and purification of PCR products**

*Cb. farciminis* KB1089 genomic DNA was isolated using a DNeasy Blood and Tissue Kit (Qiagen, Hilden, Germany) according to the manufacturer’s instructions. All plasmids constructed in this study were extracted using a QIAprep Spin Miniprep Kit (Qiagen, Hilden, Germany) and were confirmed *via* sequence analysis using a GenomeLab DTCS Quick Start Kit and a Beckman-Coulter CEQ8000 (Beckman Coulter, Brea, CA, USA). PCR amplification for cloning purposes was performed using PrimeSTAR Max DNA Polymerase (Takara Bio, Kusatsu, Japan), and colony PCR was performed using TAKARA Ex Taq (Takara Bio, Kusatsu, Japan). All oligonucleotides, used as primers for PCR (listed in Table S3) were purchased from Hokkaido System Science (Sapporo, Japan). Moreover, all PCR products were gel-purified using a QIAgen Gel Extraction Kit (Qiagen, Hilden, Germany).

**Cloning of *nukS*, *pbgS,* and *pttS* in *E. coli***

To construct pET28-*nukS*, pET28-*pbgS* and pET28-*pttS*, the *nukS*, *pbgS* and *pttS* genes were amplified from the genomic DNA of *Cb. farciminis* KB1089 by PCR using the primers oET-*nukS*-Fw/oET-*nukS*-Rv, oET-*pbgS*-Fw/oET-*pbgS*-Rv, and oET28-*pttS*-Fw/oET28-*pttS*-Rv, respectively. The PCR products were gel-purified and digested using the restriction enzymes SalI and NotI. The digested fragments were gel-purified and were ligated into the similarly digested and purified pET-28a(+) vector. The ligation products were transformed into competent *E. coli* DH5α cells using Inoue’s method^32^, and kanamycin-resistant transformants were selected. Plasmids were purified from the selected transformants. To construct pET21-*pbgS*, the *pbgS* gene was cloned into pET-21b(+), as described above, but with the selection of ampicillin-resistant transformants. Conversely, to construct pRSF-*pttS*, the *pttS* gene was cloned in the SalI and NotI sites of pRSFDuet-1, as described above. Also to construct pRSF-*pttS*-*nukS*, the *nukS* gene was amplified from the genomic DNA of *Cb. farciminis* KB1089 by PCR using the primers oRSF-*nukS*-Fw/oRSF-*nukS*-Rv. The PCR products were gel-purified and digested using the restriction enzymes BglII and XhoI. In addition, the digested fragments were gel-purified and ligated into the similarly digested and purified pRSF-*pttS* vector. The following procedures were the same as described above.

To construct *E. coli* pET28-*nukS*, *E. coli* pET28-*pbgS*, *E. coli* pET28-*pttS* and *E. coli* pRSF-*pttS-nukS*, the constructed vectors pET28-*nukS*, pET28-*pbgS*, pET28-*pttS*, and pRSF-*pttS-nukS* were, respectively, were transformed into *E. coli* Rosetta 2 (DE3)-competent cells using Inoue’s method^32^. Kanamycin- and chloramphenicol-resistant transformants were selected. Successful introduction of vectors in the selected transformants was confirmed by colony PCR using primers for the T7-promoter/T7-terminator for *E. coli* pET28-*nukS*, *E. coli* pET28-*pbgS* and *E. coli* pET28-*pttS* and primers for the ACYCDuetUP-1/T7-terminator for *E. coli* pRSF-*pttS*-*nukS*, respectively*.* To construct *E. coli* pET21-*pbgS*/pRSF-*pttS* and *E. coli* pET21*-pbgS*/pRSF-*pttS*-*nukS*, the constructed vectors pRSF-*pttS* and pRSF-*pttS*-*nukS* were simultaneously transformed with pET21-*pbgS* into *E. coli* Rosetta 2 (DE3), as described above. Ampicillin-, kanamycin- and chloramphenicol-resistant transformants were selected. Successful introduction of vectors in selected transformants was confirmed by colony PCR using the primers T7-promoter/T7-terminator and ACYCDuetUP-1/T7-terminator, respectively.

**Cloning of *nukS*, *pbgS* and *pttS* in *Lc. lactis***

To construct pNZ7021-*nukS*, pNZ7021-*pbgS*, and pNZ7021-*pttS*, the *nukS*, *pbgS* and *pttS* genes, together with approximately 100-bp upstream regions, were amplified from the genomic DNA of *Cb. farciminis* KB1089 by PCR using the primers oNZ-*nukS*-Fw/oNZ-*nukS*-Rv, oNZ-*pbgS*-Fw/oNZ-*pbgS*-Rv and oNZ-*pttS*-Fw/oNZ-*pttS*-Rv, respectively. Moreover, to construct pNZ7021-*pttS*-*pbgS*-*nukS*, the region that contained the *pttS*, *pbgS* and *nukS* genes, together with an approximately 100-bp upstream region of the start codon of the *pttS* gene, was amplified from the genomic DNA of *Cb. farciminis* KB1089 by PCR using the primers oNZ-*nukS*-Fw/oNZ-*pttS*-Rv. The PCR products were gel-purified and digested using the restriction enzymes SpeI and HindIII. The digested fragments were gel-purified and ligated into the similarly digested and purified vector pNZ7021. Then, the ligation products were transformed into competent cells of transformed *E. coli* MC1061 using Inoue’s method^32^. Chloramphenicol-resistant transformants were selected, and plasmids were purified from the transformants.

Electrocompetent cells of *Lc. lactis* NZ9000 (MoBiTec GmbH, Goettingen, Germany) were prepared according to the manufacturer’s protocol. To construct *Lc. lactis* pNZ7021-*nukS*, *Lc. lactis* pNZ7021-*pbgS*, *Lc. lactis* pNZ7021-*pttS* and *Lc. lactis* pNZ7021-*pttS*-*pbgS*-*nukS,* the constructed vectors pNZ7021-*nukS*, pNZ7021-*pbgS*, pNZ7021-*pttS* and pNZ7021-*pttS*-*pbgS*-*nukS* were transformed into electrocompetent cells of *Lc. lactis* NZ9000 by electroporation according to the manufacturer’s protocol. Chloramphenicol-resistant transformants were selected. Successful introduction of vectors in the selected transformants was confirmed by colony PCR using the primers pNZ7021-Fw/pNZ7021-Rv.

**Synthesis and analysis of sinigrin-6-sinigrin**

***Enzymatic synthesis of sinigrin-6-phosphate by BglK***

The expression vector for BglK (β-glucoside kinase from *K. pneumoniae* ATCC 23357, AAK58463.1^15^), pET28-*bglK*, was constructed using GenScript, (Piscataway, NJ, USA) and transformed into *E. coli* Rosetta 2 (DE3) as described above. The transformed cells were grown with shaking at 150 strokes min^−1^ at 37°C in 5 mL of LB medium containing 34 μg mL^−1^ chloramphenicol and 30 μg mL^−1^ of kanamycin. Subsequently, 50 μL of the overnight cultures were added to 5 mL of the same medium. IPTG was then added to a final concentration of 0.5 mM after the cultures were grown for 2.5 h with shaking at 150 strokes min^−1^ at 37°C, and cells were cultured at 37°C for 4 h with shaking at 150 strokes min^−1^. Cells were then harvested by centrifugation at 1,500 × *g* for 10 min, washed twice with 0.85% (w/v) NaCl and resuspended in 500 μL of 25 mM HEPES (pH 7.5) containing 2 mM MgSO_4_⋅7H_2_O. The cell suspensions were treated with an identical volume of glass beads (0.1 mm diameter) using a Multi-Beads Shocker MB701 (Yasui Kikai, Osaka, Japan) with six cycles at 2,700 rpm for 60 s on and 60 s off. Cell debris was removed by centrifugation at 8,000 × *g* for 20 min. The supernatants obtained were used as crude BglK.

To synthesize sinigrin-6-phosphate, 1 mL of the reaction mixture containing 350 μL of crude BglK, 3 mM sinigrin, and 1.5 mM ATP (adjusted to pH 7.5 by the addition of less than 10 μL of 1M NaOH) was incubated at 25°C for 2 h. The sugar phosphates were then extracted from the reaction mixtures after the reaction, according to the procedure described below.

***Extraction of phosphorylated sugar compounds***

Phosphorylated sugar compounds in the BglK reaction mixtures were isolated according to the procedure described previously^15^, and with some modifications. The pH in 1 mL of the samples prepared, as previously described^15^, was adjusted to 8.2 with 1 M NaOH and 0.5 mL of 25% (w/v) aqueous solution of barium acetate was added. The mixture was vortexed and chilled on ice for 30 min. Heavy white precipitates of water-insoluble Ba^2+^ salts were removed by centrifugation (1,500 × *g* for 10 min). The obtained supernatants were filtered with a membrane filter (Merck Millipore, Darmstadt, Germany) with a pore size of 0.45 μm, added to 6 mL of ethanol and chilled overnight at 4°C. The flocculent precipitates of the ethanol-insoluble Ba^2+^ salts were collected by centrifugation (1,500 × *g* for 10 min) and were dried overnight in a vacuum desiccator. The obtained pellets were stored at −20°C.

***Detection of phosphorylated sinigrin by high-performance ion chromatography–high-resolution tandem mass spectrometry (HPIC–HRMS/MS) analysis***

About 50 mg of the Ba^2+^ salts containing phosphorylated sugar compounds were redissolved in 100 μL of H_2_O, and 2 μL of the supernatant was analyzed by high-performance ion chromatography–high-resolution tandem mass spectrometry (HPIC–HRMS/MS). The HPIC was performed using the Dionex ICS-5000^+^ system (Thermo Fisher Scientific, Waltham, MA, USA) equipped with an anion electrolyte suppressor (Dionex AERS 500e 2 mm, Thermo Fisher Scientific, Waltham, MA, USA), a guard column (Dionex IonPac AG11-HC-4 μm; 3.0 mm ID × 250 mm; 4 μm particle size; Thermo Fisher Scientific, Waltham, MA, USA), and a separation column (Dionex IonPac AS11-HC-4 μm; 3.0 mm ID × 250 mm; 4 μm particle size; Thermo Fisher Scientific, Waltham, MA, USA). The HPIC flow rate was 0.3 mL min^–1^, supplemented post-column with 0.1 mL min^–1^ makeup flow of 1 mM ammonium acetate in MeOH. The gradient conditions for the KOH eluent for HPIC separation were set as follows: 0.0–24.0 min, a linear gradient from 10 mM to 100 mM; 24.0–27.0 min, 100 mM; 27.0–27.1 min, a linear gradient from 100 mM to 10 mM; 27.1–30 min, 10 mM.

HRMS/MS was performed using a Q Exactive, which is a high-performance benchtop quadrupole-Orbitrap mass mass spectrometer (Thermo Fisher Scientific, Waltham, MA, USA), equipped with a heated under an electrospray ionization source. The conditions of the HRMS analysis were as follows: polarity, negative ionization; sheath gas flow rate, 40 arb; auxiliary gas flow rate, 10 arb; spray voltage, 2.0 kV; capillary temperature, 350°C; S-lens level, 50; probe heater temperature, 300°C; mass resolution, 70,000; automatic gain control target (the number of ions to fill the C-trap), 100,000; maximum injection time, 100 ms and MS scan range, 70–1,050 (m/z). The parallel reaction-monitoring conditions for each target compound were as follows: mass resolution, 70,000; automatic gain control target, 200,000; maximum injection time, 100 ms; isolation window, 4.0 (m/z); and stepped normalized collision energy, 10, 20, and 35.

**Supplementary reference**

32. Inoue, H., Nojima, H. & Okayama, H. High efficiency transformation of *Escherichia coli* with plasmids. *Gene* **96**, 23–28 (1990).

Fig. S1. GC-MS analysis of the sinigrin converted products by washed cells of *Cb. farciminis* KB1089. A, A GC-MS total ion current chromatogram of 3 mM AITC in 20 mM KPB (pH 6.5). B, The mass spectrum at the retention time 3.802 min in the GC-MS chromatogram A. C, A GC-MS total ion current chromatogram of the sinigrin converted products in the resting cell reaction using washed cells of *Cb. farciminis* KB1089. D, A mass spectrum at the retention time 3.811 min in the GC-MS chromatogram C.

**Fig. S2. HPIC-HRMS/MS analysis of sinigrin converted products (phosphorylated sinigrin) by cell-free extracts of *E. coli* pET28-*bglK*. A,** HPIC-MS chromatograms at *m/z* 437.9935 of the sinigrin converted products by cell-free extracts of *E. coli* pET28-*bglK* (I) and the vector control strain, *E. coli* pET-28a(+) (II). **B**, Expected fragmentation patterns of sinigrin-6-phosphate as a representative of sinigrin monophosphate. **C**, HRMS/MS spectra at the retention time 24.92 min in HPIC-HRMS chromatograms **A**-(I). **D**, HRMS/MS spectra at the retention time 26.31 min in HPIC-HRMS chromatograms **A**-(I).

**Fig. S3. GC-MS analysis of a sinigrin monophosphate converted product (AITC) by cell-free extracts of *Lc. lactis* pNZ7021-*pbgS*. A,** GC-MS SIM chromatograms at *m/z* 99 of the reaction products by *Lc. lactis* pNZ7021-*pbgS* with the sinigrin converted products by cell-free extracts of *E. coli* pET28-*bglK* (yellow) and *E. coli* pET-28a(+) (black) as the reaction substrates. **B**, The difference mass spectrum at the retention time of 3.77 min obtained by subtracting the mass spectrum of the reaction products by *Lc. lactis* pNZ7021-*pbgS* with the sinigrin converted products by cell-free extracts of *E. coli* pET-28a(+) from the mass spectrum of the reaction products by *Lc. lactis* pNZ7021-*pbgS* with the sinigrin converted products by cell-free extracts of *E. coli* pET28-*bglK.*The experiments were performed in triplicate. The molecular weight of AITC is 99.15.

**Table S1. Bacterial strains used in this study.**

| **Strain** | **Description** | **Source or reference** |
| --- | --- | --- |
| *Cb. farciminis* KB1089 | Isolated from pickled Japanese turnips | This study |
| *Cb. farciminis* KCTC3681  (= JCM1092 = DSM20184) | Able to metabolize sinigrin | JCM |
| *Cb. farciminis* LMG9200  (= DSM20184) | Able to metabolize sinigrin | LMG |
| *Cb. farciminis* NRIC0417 | Able to metabolize sinigrin | NRIC |
| *Cb. farciminis* LMG9189 | Unable to metabolize sinigrin | LMG |
| *Cb. farciminis* NRIC0416 | Unable to metabolize sinigrin | NRIC |
| *E. coli* DH5α | Commercial transformation host for all plasmids except for pNZ7021 series | TOYOBO |
| *E. coli* MC1061 | Commercial transformation host for pNZ7021 series | MoBiTec |
| *E. coli* Rosetta 2 (DE3) | Commercial expression host | Novagen^®^ |
| *E. coli* pET-28a(+) | *E. coli* Rosetta 2 (DE3) harboring pET-28a(+) | This study |
| *E. coli* pET-21b(+)/pET-28a(+) | *E. coli* Rosetta 2 (DE3) harboring pET-21b(+) and pET-28a(+) | This study |
| *E. coli* pRSFDuet-1 | *E. coli* Rosetta 2 (DE3) harboring pRSFDuet-1 | This study |
| *E. coli* pET-21b(+)/pRSFDuet-1 | *E. coli* Rosetta 2 (DE3) harboring pET-21b(+) and  pRSFDuet-1 | This study |
| *E. coli* pET28-*nukS* | *E. coli* Rosetta 2 (DE3) harboring pET-*nukS* | This study |
| *E. coli* pET28-*pbgS* | *E. coli* Rosetta 2 (DE3) harboring pET-*pbgS* | This study |
| *E. coli* pET28-*pttS* | *E. coli* Rosetta 2 (DE3) harboring pET-*pttS* | This study |
| *E. coli* pRSF-*pttS*-*nukS* | *E. coli* Rosetta 2 (DE3) harboring pRSF-*pttS*-*nukS* | This study |
| *E. coli* pET21-*pbgS*/pET28-*nukS* | *E. coli* Rosetta 2 (DE3) harboring pET-*pbgS* and pET-*nukS* | This study |
| *E. coli* pET21-*pbgS*/pRSF-*pttS* | *E. coli* Rosetta 2 (DE3) harboring pET-*pbgS* and pRSF-*pttS* | This study |
| *E. coli* pET21-*pbgS*/pRSF-*pttS*-*nukS* | *E. coli* Rosetta 2 (DE3) harboring pET-*pbgS* and  pRSF-*pttS*-*nukS* | This study |
| *E. coli* pET28-*bglK* | *E. coli* Rosetta 2 (DE3) harboring pET-*bglK* | This study |
| *Lc. lactis* NZ9000 | Derivative of *Lc. lactis* subsp. *cremoris* MG1363, *pepN*::*nisRK* | MobiTec |
| *Lc. lactis* pNZ7021 | *Lc. lactis* harboring pNZ7021 | This study |
| *Lc. lacits* pNZ7021-*nukS* | *Lc. lactis* harboring pNZ7021-*nukS* | This study |
| *Lc. lacits* pNZ7021-*pbgS* | *Lc. lactis* harboring pNZ7021-*pbgS* | This study |
| *Lc. lactis* pNZ7021-*pttS* | *Lc. lactis* harboring pNZ7021-*pttS* | This study |
| *Lc. lacits* pNZ7021-*pttS*-*pbgS*-*nukS* | *Lc. lactis* harboring pNZ7021-*pttS*-*pbgS*-*nukS* | This study |

**Table S2. Plasmids used in this study.**

| **Plasmid** | **Description** | **Source** |
| --- | --- | --- |
| pET-28a(+) | Kan^R^, T7 promoter, pBR322ori, expression vector | Novagen, Inc. |
| pET-21b(+) | Amp^R^, T7 promoter, pBR322ori, expression vector | Novagen, Inc. |
| pRSFDuet-1 | Kan^R^, T7 promoter, RSFori, co-expression vector | Novagen, Inc. |
| pET28-*nukS* | Derivative of pET-28a(+) in which *nukS* gene  was cloned in SalI and NotI sites | This study |
| pET28-*pbgS* | Derivative of pET-28a(+) in which *pbgS* gene  was cloned in SalI and NotI sites | This study |
| pET28-*pttS* | Derivative of pET-28a(+) in which *pttS* gene  was cloned in SalI and NotI sites | This study |
| pET28-*bglK* | Derivative of pET-28a(+) in which *bglK* gene encoding *β*-glucoside kinase (AAK58463.1)  was cloned in SalI and NotI sites | This study |
| pET21-*pbgS* | Derivative of pET-21b(+) in which *pbgS* gene  was cloned in SalI and NotI sites | This study |
| pRSF-*pttS* | Derivative of pRSFDuet-1 in which *pttS* gene  was cloned in SalI and NotI sites | This study |
| pRSF-*pttS*-*nukS* | Derivative of pRSF-*pttS* in which *nukS* gene  was cloned in BglII and XhoI sites | This study |
| pNZ7021 | Cm^R^, P_pepN_ promoter (constitutive promoter), *repC*, *repA*, expression vector for *Lc. lactis* NZ9000 | MobiTec Co., Ltd. |
| pNZ7021-*nukS* | Derivaive of pNZ7021 in which *nukS* gene  was cloned in SpeI and HindIII sites | This study |
| pNZ7021-*pbgS* | Derivative of pNZ7021 in which *pbgS* gene  was cloned in SpeI and HindIII sites | This study |
| pNZ7021-*pttS* | Derivative of pNZ7021 in which *pttS* gene  was cloned in SpeI and HindIII sites | This study |
| pNZ7021  -*pttS*-*pbgS*-*nukS* | Derivative of pNZ7021 in which *pttS*, *pbgS*, and *nukS* genes were tandemly cloned in SpeI and HindIII sites | This study |

**Table S3. Oligonucleotides used in this study.** The underlined sequence represents restriction sites.

| **Oligonucleotide** | **Sequence (5’ to 3’)** |
| --- | --- |
| 27F | AGAGTTTGATCCTGGCTCAG |
| 1525R | AAGGAGGTGATCCAGCC |
| 516F | TGCCAGCAGCCGCGGTA |
| 1087F | GGTTAAGTCCCGCAACGA |
| 531R | TACCGCGGCTGCTGGCA |
| 1104R | TCGTTGCGGGACTTAACC |
| 518R | GTATTACCGCGGCTGCTGG |
| pheS-21F | CCGCAGCCAA |
| pheS-22R | AGGTGACCGT |
| oET-*nukS*-Fw | CCGGTCGACATGAGTAATATAACATTAGTTAACAGTGAA |
| oET-*nukS*-Rv | CCGAGATCTATGAGTAATATAACATTAGTT |
| oET-*pbgS*-Fw | CCGGTCGACATGAAATTTCCAAAGAACTTTTTATGGGGC |
| oET-*pbgS*-Rv | TTCGCGGCCGCTCATTTTACATATGCTCCTTGCAATTCATT |
| oET-*pttS*-Fw | CCGGTCGACATGAGTAAAAATCAGCTCGCAAAATTAATT |
| oET-*pttS*-Rv | TTCGCGGCCGCTCAGGCAATGTTCAATAATGAATCGCCAAC |
| oRSF-*nukS-*Fw | TTCGCGGCCGCTCAATCACGTTCAACATCTCCGTAACGGCC |
| oRSF-*nukS-*Rv | TTCCTCGAGTCAATCACGTTCAACATCTCC |
| oNZ-*nukS*-Fw | CCGACTAGTGGACGTGGAACTGGAAATAG |
| oNZ-*nukS*-Rv | TTCAAGCTTTCAATCACGTTCAACATCTC |
| oNZ-*pbgS*-Fw | CCGACTAGTAGTCACAAATAGTAATGAT |
| oNZ-*pbgS*-Rv | CCGACTAGTAGTCACAAATAGTAATGAT |
| oNZ-*pttS*-Fw | CCGACTAGTTGAGATGTTGCTGCAACAC |
| oNZ-*pttS*-Rv | TTCAAGCTTTCAGGCAATGTTCAATAATG |
| T7-promoter | TAATACGACTCACTATAGGG |
| T7-terminator | ATGCTAGTTATTGCTCAGCGG |
| ACYCDuetUP-1 | GGATCTCGACGCTCTCCCT |
| pNZ7021-Fw | AGATCTGTCGACCTGCAGTA |
| pNZ7021-Rv | TCAACTGCTGCTTTTTGGCT |

**Table S4. InterPro analysis of PttS, PbgS and NukS.**

|  | InterPro Entry type | Amino acid residue | Description | InterPro ID |
| --- | --- | --- | --- | --- |
| PttS | Family | 4-626 | Phosphotransferase system, beta-glucoside-specific IIABC component | IPR011297 |
|  | Domain | 4-86 | Phosphotransferase system, IIB component, type 1 | IPR001996 |
|  |  | 103-461 | Phosphotransferase system, EIIC component, type 1 | IPR013013 |
|  |  | 480-604 | Phosphotransferase system, sugar-specific permease EIIA type 1 | IPR001127 |
|  |  |  |  |  |
| PbgS | Family | 2-469 | Glycoside hydrolase family 1 |  |
|  | Domain | not assigned | - | - |
|  |  |  |  |  |
| NukS | Family | not assigned | - | - |
|  | Domain | not assigned | - | - |
|  | Homologous Superfamily | 3-193 | P-loop containing nucleoside triphosphate hydrolase | IPR027417 |
